# Nondestructive label-free detection of peritumoral white matter damage using cross-polarization optical coherence tomography

**DOI:** 10.3389/fonc.2023.1133074

**Published:** 2023-03-02

**Authors:** Ksenia A. Achkasova, Alexander A. Moiseev, Konstantin S. Yashin, Elena B. Kiseleva, Evgenia L. Bederina, Maria M. Loginova, Igor A. Medyanik, Grigory V. Gelikonov, Elena V. Zagaynova, Natalia D. Gladkova

**Affiliations:** ^1^ Research institute of experimental oncology and biomedical technologies, Privolzhsky Research Medical University, Nizhny Novgorod, Russia; ^2^ Laboratory of Highly Sensitive Optical Measurements, Institute of Applied Physics of Russian Academy of Sciences, Nizhny Novgorod, Russia; ^3^ Department of oncology and neurosurgery, University clinic, Privolzhsky Research Medical University, Nizhny Novgorod, Russia; ^4^ Department of pathology, University clinic, Privolzhsky Research Medical University, Nizhny Novgorod, Russia; ^5^ Lobachevsky State University, Nizhny Novgorod, Russia

**Keywords:** optical coherence tomography, peritumoral white matter, myelin, brain tumor, neurosurgery, attenuation coefficient

## Abstract

**Introduction:**

To improve the quality of brain tumor resections, it is important to differentiate zones with myelinated fibers destruction from tumor tissue and normal white matter. Optical coherence tomography (OCT) is a promising tool for brain tissue visualization and in the present study, we demonstrate the ability of cross-polarization (CP) OCT to detect damaged white matter and differentiate it from normal and tumor tissues.

**Materials and methods:**

The study was performed on 215 samples of brain tissue obtained from 57 patients with brain tumors. The analysis of the obtained OCT data included three stages: 1) visual analysis of structural OCT images; 2) quantitative assessment based on attenuation coefficients estimation in co- and cross-polarizations; 3) building of color-coded maps with subsequent visual analysis. The defining characteristics of structural CP OCT images and color-coded maps were determined for each studied tissue type, and then two classification tests were passed by 8 blinded respondents after a training.

**Results:**

Visual assessment of structural CP OCT images allows detecting white matter areas with damaged myelinated fibers and differentiate them from normal white matter and tumor tissue. Attenuation coefficients also allow distinguishing all studied brain tissue types, while it was found that damage to myelinated fibers leads to a statistically significant decrease in the values of attenuation coefficients compared to normal white matter. Nevertheless, the use of color-coded optical maps looks more promising as it combines the objectivity of optical coefficient and clarity of the visual assessment, which leads to the increase of the diagnostic accuracy of the method compared to visual analysis of structural OCT images.

**Conclusions:**

Alteration of myelinated fibers causes changes in the scattering properties of the white matter, which gets reflected in the nature of the received CP OCT signal. Visual assessment of structural CP OCT images and color-coded maps allows differentiating studied tissue types from each other, while usage of color-coded maps demonstrates higher diagnostic accuracy values in comparison with structural images (F-score = 0.85-0.86 and 0.81, respectively). Thus, the results of the study confirm the potential of using OCT as a neuronavigation tool during resections of brain tumors.

## 1 Introduction

Malignant tumors are recognized to be the second leading cause of human deaths around the world with more than 250 000 deaths caused by brain tumors annually (1). The most common neoplasms of the brain are malignant gliomas developing from neuroglial cells (astrocytes, oligodendrocytes) (2). The main paradigm for treating patients with brain gliomas remains achieving the maximum possible life expectancy while at the same time maintaining its high quality.

Despite the improvements in the diagnosis and treatment of brain neoplasms, however, tumor development often leads to the onset of neurological deficit, which is primarily associated with its infiltrative growth into the surrounding white matter. The invasion of tumor cells into the white matter leads to morphological and functional changes in myelinated fibers in the peritumoral region, resulting in a violation of the nerve impulse conduction (3, 4). Moreover, different treatment options may also cause various pathological changes in brain tissues. For example, accidental damage to the healthy white matter pathways may occur during tumor resection due to the absence of a method allowing performing intraoperative assessment of white matter morphological features in the peritumoral area (5).

In view of the leading role of the white matter in organizing the integral functionality of the brain and its low degree of plasticity (that is, the low ability to recover) in comparison with the cerebral cortex, the study of its morphological and functional changes in glial brain tumors and their treatment is an important scientific and practical task. The ability to intraoperatively determine and predict the degree of damage to the white matter can increase the quality of tumor resections, allow more accurate choice of chemo- and radiotherapy treatment regimens and development of drugs to protect the brain during combined treatment.

Diffusion-tensor MRI (DT-MRI) is currently the only method for *in vivo* evaluation of the state of the brain pathways, which can qualitatively assess the relative position of the tumor and fiber tracts and quantitatively evaluate orientation and preservation of the nerve pathways (6, 7). In case of the glial tumor surgery, DT-MRI imaging allows the surgeon to select a surgical approach to the tumor focus, bypassing healthy pathways (8). However, the limitation of this method is its insufficient resolution and the impossibility of intraoperative use for analyzing the white matter structure in the exact region of interest due to possible discrepancies between MRI images and real situation caused by displacement of brain structures as a result of intracranial pressure during tumor resection (known as “brain shift”) (9).

Several intraoperative imaging techniques are available at present to visualize tumor tissue by detecting its typical features (10, 11), however they do not make it possible to intraoperatively detect the damage of the white matter in the peritumoral region. Carrying out neurosurgical intervention using only these methods may lead, on the one side, to damage of the healthy brain tissues and on the other side, to leaving areas with destroyed nerve fibers. The persistence of these non-viable areas, abundantly infiltrated with tumor cells, is likely to lead to early tumor recurrence. Therefore, all the abovementioned emphasizes the need for developing new methods of intraoperative imaging of brain tissues being able to address current demands.

Optical coherence tomography (OCT) is a rapidly developing, minimally invasive, label-free method for real time visualizing the structure of biological tissues with a resolution up to a few micrometers at a depth up to 1.5 mm (12). Currently, OCT occupies a leading place in the clinical practice among optical diagnostic methods due to its high resolution, high speed of obtaining and evaluating images, as well as the availability of several modalities. Several studies have shown the promise of using OCT to differentiate between normal and tumorous white matter with high diagnostic accuracy (13, 14). Targeted visualization of white matter, including individual myelinated fibers, is possible with the use of special functional extensions of OCT - polarization-sensitive OCT (PS OCT) (15) and cross-polarization OCT (CP OCT) (13), sensitive to the phenomenon of light birefringence in tissues which allows obtaining high-quality information about the presence of elongated structures (myelinated fibers).

Identifying scattering, and, accordingly, morphological, features of biological tissues using the OCT method is based on two main approaches to the analysis of OCT images: qualitative (visual) and quantitative (16–18). Qualitative approach is based on visual analysis of structural two-dimensional OCT images, which is considered subjective. However, this method is still the only one available for use in clinical practice in several countries (19). The quantitative approach to the analysis of OCT images is based on the calculation of optical coefficients, where the attenuation coefficient is frequently used for this purpose (14, 16, 20). This method allows objectifying the obtained data, which leads to a decrease in the influence of the “human factor” on the interpretation of the results. The use of color-coded maps that display the distribution of coefficient values throughout the image allows one to combine the advantages of both approaches, namely, the clarity of the visual method and the objectivity of the quantitative one.

In the present work, for the first time we study the scattering properties of peritumoral white matter, characterized by the destruction of myelinated fibers, and determine the diagnostic ability of CP OCT to differentiate three brain tissue types in the peritumoral area (white matter, damaged white matter and tumor) based on the qualitative and quantitative processing of OCT images.

## 2 Materials and methods

### 2.1 Ex vivo studies of the human brain specimens

The study was carried out on *ex vivo* samples of brain tissue obtained during tumor resection from 57 patients with brain neoplasms aged from 36 to 60 including 52 patients with gliomas of different degrees of malignancy (astrocytoma Grade I-II (n = 18), astrocytoma Grade III (n = 15), glioblastoma Grade IV (n = 19)) and 5 patients with brain metastasis of extracerebral tumors (see **Table 1**). A surgical approach to tumor node was carried out in accordance with the plan of surgical intervention without any changes due to sample collection. During access to the tumor node, white matter that is usually exposed to resection or coagulation during surgery was removed. Tissue sampling included two main stages: at the first stage, samples of normal white matter were obtained, as far as possible from the tumor; at the second stage, white matter sampling was carried out at the direct adjacency to the tumor node. In addition, samples from tumor core were collected. In several cases it was possible to obtain samples that included both tumor and peritumoral white matter.

**Table 1:** The characteristics of patients included in the study.

Number of patients	57
Sex (male/female)	35/22
Age (average (min – max))	47 (36 – 60)
Tumor Grade (I-II/III/IV/metastasis)	18/15/19/5
Tumor localization (frontal lobe/parietal lobe/temporal lobe/occipital lobe)	31/14/27/6

Each sample was immediately placed in Petri dish, covered with cotton cloth moistened with saline solution, closed to prevent dehydration, put on ice and delivered to the location of CP OCT study, which took several minutes. To create a fresh flat surface, each sample was cut proximately before the CP OCT study. The CP OCT study was performed in a contactless mode when the sample was placed on a special motorized table under the OCT probe, which took 10-15 minutes for each sample. In total, 158 samples of normal and peritumoral white matter and 98 tumor samples were scanned; 930 CP OCT images of white matter and 400 images of tumor were obtained. One can consider each patient as a separate data point from the sample and sampling several images from each patient’s data can be considered as a Kernel Density Estimation (KDE) which can provide an estimate of the density distribution of the attenuation coefficient from the limited number of measured data points (21). It is necessary to mention that during subsequent analysis of morphological features and CP OCT images of tumors we did not divide them into subgroups according to the Grade or “glioma/metastasis”. All kinds of tumors were included into one group and analyzed altogether.

The study was approved by Institutional Review Board of Privolzhsky Research Medical University and informed consent was obtained from all patients. All the methods were performed in accordance with the relevant guidelines and regulations.

### 2.2 Histological analysis

After imaging, all of the samples were forwarded to histological study. The scanning area of each sample was marked with histological ink; the sample was fixed in 10% formalin for 48 hours. The ink mark was used as a guide for the subsequent match of the histological sections and en-face CP OCT images. Histological sections of tumors and white matter were stained with hematoxylin & eosin (H&E) to obtain general information about the sample. White matter samples were selected for further study, samples containing tumor or gray matter areas were excluded from this stage of the study. For targeted study of samples of white matter, sections were stained using Luxol fast blue with crezyl violet to identify myelinated fibers structure. With this staining, the preserved myelin sheath is stained bright blue, which makes it possible to assess the morphology of the fibers. Violation of the structure of myelin, which occurs due to various reasons, leads to the absence of staining of the fibers. All sections were studied by the pathologist using light microscopy and classified into three main tissue types: 1) normal white matter (numerous preserved densely packed myelinated fibers are visualized), n=41; 2) damaged white matter (loss of fiber staining is observed, ≥20% of fibers are damaged and infiltration of tumor cells is observed), n=76; 3) tumor, n=98.

### 2.3 CP OCT device

The study was performed with a spectral-domain CP OCT device (Institute of Applied Physics of Russian Academy of Sciences, Nizhny Novgorod, Russia). The system has a common-path interferometric layout operating on 1310 nm central wavelength. CP characterizes the change in polarization state due to propagation in anisotropic media. The active polarization control system is based on the analysis of the polarization state of the light returned from the tip of the optical probe. The axial resolution is 10 µm and transverse resolution is 15 µm in air. The utilized device has a 20000 A-scans/s scanning rate and performs lateral scanning of 2,4x2,4 mm^2^ (256x256 A-scans) area to obtain a backscattered light distribution in the polarization with the same and reversed rotations of the electric-field vector (22).

### 2.4 CP OCT data evaluation

Before analyzing the obtained OCT data, we selected artifact-free images (cropped and damaged images were excluded). As a result, the study included 576 white matter OCT images and 297 tumor OCT images.

**Figure 1:**
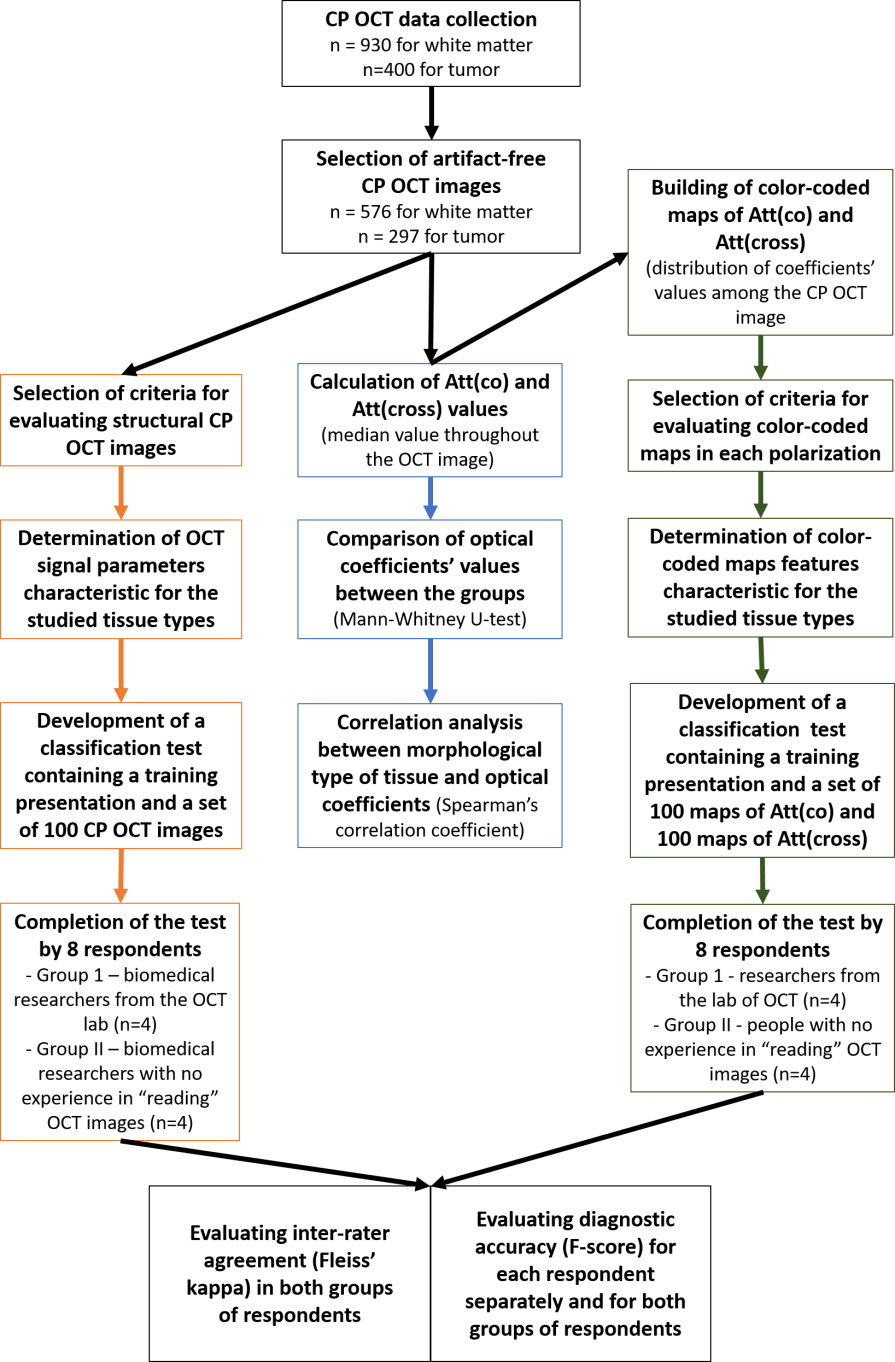
CP OCT study design.

The analysis of the obtained CP OCT data was carried out in three stages (**Figure 1**). At the first stage, a visual analysis of structural CP OCT images was performed to identify signal parameters distinctive for each studied tissue type. After that, a test was compiled, consisting of a training presentation and a set of 100 CP OCT images, offered to respondents for evaluation. The test was offered to 8 blinded researchers, including 4 researchers from the laboratory of optical coherence tomography who work daily with OCT images (Group 1) and 4 biomedical researchers with no previous experience in “reading” OCT images (Group 2). Based on the analyzed signal parameters, the respondents had to classify the images into one of three types of tissue and assign a number to each image based on the intended tissue type, where: 1 – normal white matter, 2 – damaged white matter, 3 – tumor. The first stage is described in detail in section 2.4.1. At the second stage, described in section 2.4.2.1, for each CP OCT image, the attenuation coefficients in co- and cross-polarizations were calculated with obtaining the median values of the coefficients for each image, followed by comparison between tissue types and correlation analysis of tissue types and attenuation coefficients values. The third stage described in section 2.4.2.2 included building color-coded maps of the distribution of attenuation coefficients values with subsequent visual analysis. This stage was carried out on the same arrays of CP OCT data as the first one. For each type of tissue, characteristic visual features of optical maps were identified in co- and cross polarizations. A second classification test was then compiled containing a training presentation, a set of 100 Att(co) maps and 100 Att(cross) maps. This test was also offered to the same groups of respondents. Based on the results of two tests, the level of inter-rater agreement in each group was identified using the Fleiss’ kappa, and the level of diagnostic accuracy was calculated using the F-score parameter (for each respondent separately, as well as for both groups of respondents).

#### 2.4.1 Qualitative analysis

A qualitative (visual) analysis of two-dimensional CP OCT images was carried out to study the nature of the OCT signal received from different types of tissue and to develop criteria for their differentiation. A structural CP OCT image consists of B-scans in co- (upper part of the image) and cross-polarizations (lower part of the image) stitched together. During visual assessment, we performed a complex analysis of OCT signal in both polarizations.

Based on the results of previous studies (23), we chose the following parameters for evaluating structural images and their subsequent classification:

1) OCT signal intensity (intense/non-intense) – characterizes OCT signal level throughout the image

2) OCT signal attenuation rate (low/high) - characterizes the degree of penetration of the probing radiation over the depth of the object

3) Uniformity of OCT signal attenuation (uniform/non-uniform) - characterizes the variability of the penetration depth of the probing radiation in different parts of the OCT image.

For each studied tissue type, distinctive OCT signal features were evaluated. Then, to determine the diagnostic ability of visual analysis of CP OCT images to differentiate between three types of tissue (normal white matter, damaged white matter and tumor), a special test was developed containing a training set of images demonstrating classification criteria (**Supplementary Figure 1**), typical images of each tissue type (**Supplementary Figure 2**) and the test itself of 100 images (30 images of normal white matter, 40 images of damaged white matter and 30 tumor images). For each image included in the test, three response options were indicated: “normal white matter”, “damaged white matter” and “tumor”. As it was mentioned above, the test was offered to 8 blinded researchers, divided into two groups.

#### 2.4.2 Quantitative analysis

##### 2.4.2.1 Calculation of the attenuation coefficient

To quantify the optical properties of brain tissue relying on OCT data, we used attenuation coefficients in co- (Att(co)) and cross-polarization (Att(cross)) modes. We expect that the additional usage of Att(cross) may provide us with more information about the morphological features of the white matter.

Depth-resolved approach was applied for the quantitative assessment of the OCT data in co-polarization. Such an approach was proposed in (24) under the assumption that the backscattering coefficient is proportional to the attenuation coefficient with the constant ratio between the two in the OCT depth range:


(1) $$I_{i}∼\alpha \cdot \mu_{att}(z_{i})\cdot \exp\left[-2\cdot \sum_{j=0}^{i}\mu_{att}(z_{i})\cdot \Delta \right]$$

where I*_i_* is the sum of OCT signal intensities in both polarization channels, µ*_att_* is the specimen attenuation coefficient, z*_i_* is the depth coordinate, Δ is the pixel size along the axial dimension.

In the present study, the method from (25) was adopted since it accounts for the noise with the non-zero mean, present in the distributions of the measured absolute values of the OCT images and allows to avoid systemic attenuation coefficient estimation bias, characteristic for the (24). According to (25), the depth-resolved attenuation coefficient can be written as:


(2) $$\begin{array}{l} \mu_{i}=\frac{H_{i}\cdot SNR_{i}^{\mu }}{{\left|H_{i}\right|}^{2}\cdot SNR_{i}^{\mu }+1}\cdot \mu_{i}^{est}\\ H_{i}=1-\frac{\sum_{i+1}^{\infty }N_{j}}{\sum_{i+1}^{\infty }I_{j}+\sum_{i+1}^{i_{\max}}N_{j}}=1-\frac{\langle N\rangle \cdot (i_{max}-i)}{\sum_{i+1}^{\infty }I_{j}+\sum_{i+1}^{i_{\max}}N_{j}}\\ SNR_{i}^{\mu }=\sum_{x_{i},z_{i}\in W}\frac{{\left|\mu_{i}^{est}\right|}^{2}-{\left|N_{i}^{\mu }\right|}^{2}}{{\left|N_{i}^{\mu }\right|}^{2}}\\ N_{i}^{\mu }=\frac{N_{i}}{2\Delta \sum_{i+1}^{i_{\max}}\left(I_{j}+N_{j}\right)}=\frac{\langle N\rangle }{2\Delta \sum_{i+1}^{i_{\max}}\left(I_{j}+N_{j}\right)} \end{array}$$

where <N > is the amplitude of the noise floor, which can be estimated before the measurements, SNR*_i_^µ^* is the local signal-to-noise ratio (SNR) for the attenuation coefficient distribution, which is estimated by the averaging in the rectangular window with the side of W pixels. The W value should be sufficiently large (≥32 pixels) to provide sufficient statistics inside each window. The value (I*_j_* +N*_j_*) is simply the measured signal at the depth *j.* Thus, all the values from Eq. (2) can be measured from the cross-sectional OCT intensity distributions. According to (25), the confocality and the spectral roll-off for the OCT system used in the study will lead to the attenuation coefficient estimation error which will not exceed 10%, thus these factors were not considered in the present study.

To calculate Att(cross) values, the method of linear fitting of the logarithmic signal described in (14) was used because the differential equations describing signal propagation in co-polarization are not valid for the signal in cross-polarization, and, consequently, the method based on their solution cannot be directly applied to a signal in cross-polarization.

Both attenuation coefficients were estimated in the depth range of 120–300 µm. The choice of depths was determined by the construction of the most contrasting color-coded maps in this range, providing the best information about the morphology of brain tissue.

##### 2.4.2.2 En-face color-coded map building

En-face color-coded maps were constructed based on the distribution of coefficients values for each OCT image in co- and cross-polarizations. Based on the range of the numerical values of the optical coefficients for the studied tissue types, a universal color scale was selected for maps in co- and cross-polarization, which allows differentiating brain tissues in the specified color range. Visual criteria for color-coded maps in both polarizations corresponding to the studied three types of tissue were determined, namely, the predominance of one or another color, the color variation in maps (**Supplementary Figure 3**). Then a special test was developed containing a training set and a set of 100 color-coded maps of each attenuation coefficient (30 maps of normal white matter, 40 maps of damaged white matter and 30 maps of tumor). This test was also offered to two groups of blinded researchers mentioned above.

Although the selected rainbow colormap 'jet' is widely criticized for its poor performance, since small variations in the color green are not perceived as green is a common natural color, while small variations in the colors red and blue are perceived (26, 27), the difference between the attenuation coefficient values for the tumorous and normal tissues allows assigning colors of maximal contrast (i.e. red and blue) for the two classes of interest, which lead to the easy visual differentiation of these classes for the user.

#### 2.4.3 Statistical analysis

Statistical analysis was performed using GraphPad Prism 8 and SPSS Statistics 26. According to the results of the test aimed at classifying OCT images according to visual criteria of B-scans and color-coded maps, we calculated the inter-rater agreement level and F-score. Inter-rater agreement was calculated using the Fleiss’ kappa (k) coefficient: k ≥ 0.8 – perfect agreement; 0.7≥ k < 0.8 – substantial agreement; k<0.7 – poor agreement. F-score represents the measure of a test’s accuracy in case of a multiclass classification. Its values were interpreted in the following way: f >0.9 – excellent diagnostic accuracy, 0.8< f ≤0.9 – good, 0.5< f ≤0.8 – fair, f<0.5 – poor. To evaluate the results of quantitative image processing, we used the median value among all values of every optical coefficient calculated for each A-scan of 3D CP OCT image. The results are expressed as Me [Q1;Q3], where Me – is the median value of optical coefficient; Q1, Q3 – are the values of 25th and 75th percentiles, respectively. To compare optical coefficient values of different tissue types, we used the Mann-Whitney U-test with the hypothesis that there was no difference between the compared groups. To establish correlation level between optical coefficients values and studied brain tissue types we calculated the Spearman’s correlation coefficient.

## 3 Results

### 3.1 Visual assessment of CP OCT images of normal white matter, damaged white matter and tumor tissue

**Figure 2:**
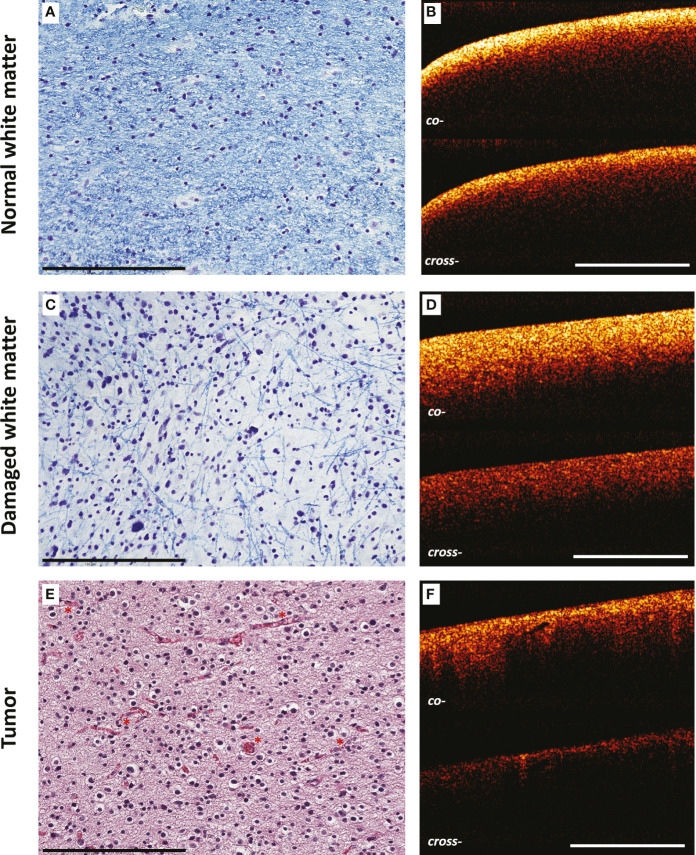
Comparison of morphological features of brain tissues and CP OCT signal. Histological images of normal **(A)** and damaged **(B)** white matter stained by Luxol fast blue, tumor tissue **(C)** (astrocytoma Grade III) stained by H&E and corresponding CP OCT images **(D–F)**, respectively. The destruction of myelinated fibers in peritumoral area is reflected in histological images (only individual preserved fibers are visualized in comparison with normal white matter) **(B)** and leads to the changes in CP OCT signal: slight reduction of the signal intensity and decrease in the attenuation rate in both polarizations **(E)**. Fundamentally different structural characteristics of the tumor, expressed in the predominance of cellular elements, the presence of a large number of blood vessels (marked by red asterisks), are reflected in a decrease in the intensity of the OCT signal in both polarizations and its heterogeneity **(F)**. Scale bar = 200 µm on histological image and 1 mm on OCT image.

This part of the study was devoted to the analysis of structural CP OCT images and determination of OCT signal parameters specific for studied tissue types. We found that the white matter on ex vivo CP OCT images is characterized by the following features: 1) high intensity of the OCT signal in both polarizations; 2) high attenuation rate of the OCT signal in both polarizations; 3) homogeneity of the attenuation of the OCT signal in both polarizations. The features of the OCT signal received from the tumor tissue are opposite, namely: 1) low intensity of the OCT signal in both polarizations; 2) low attenuation rate of the OCT signal in both polarizations; 3) inhomogeneity of the attenuation of the OCT signal in both polarizations. The variation in the scattering properties of normal and tumorous white matter tissues are due to differences in the structural characteristics of these types of tissues. The white matter contains a large number of myelinated fibers with highly scattering properties (**Figure 2A**). The tumor, on the other hand, contains mainly cellular elements, which results in a low attenuation of the probing radiation (**Figure 2C**). In the case of damaged white matter, the nature of the received OCT signal changes: there is a decrease in the signal intensity in both polarizations (while the signal in cross-polarization decreases more significantly) in comparison with healthy white matter (however, it remains higher than in the tumor), as well as a decrease in the signal attenuation rate in both polarizations (**Figure 2D**). In rare cases, signal attenuation inhomogeneity may be observed, however, such cases are an exception, and it is not clear which morphological features cause these changes of OCT signal. Histologically, damaged white matter is characterized by destruction of myelinated fibers while only individual preserved fibers can be visualized as well as infiltration of tumor cells (**Figure 2C**).

Thus, each of the three types of tissue has a unique combination of OCT signal characteristics, which indicates the validity of using visual analysis of CP OCT images to differentiate the studied tissues.

### 3.2 Quantitative assessment of CP OCT images of normal white matter, damaged white matter and tumor tissue

**Figure 3:**
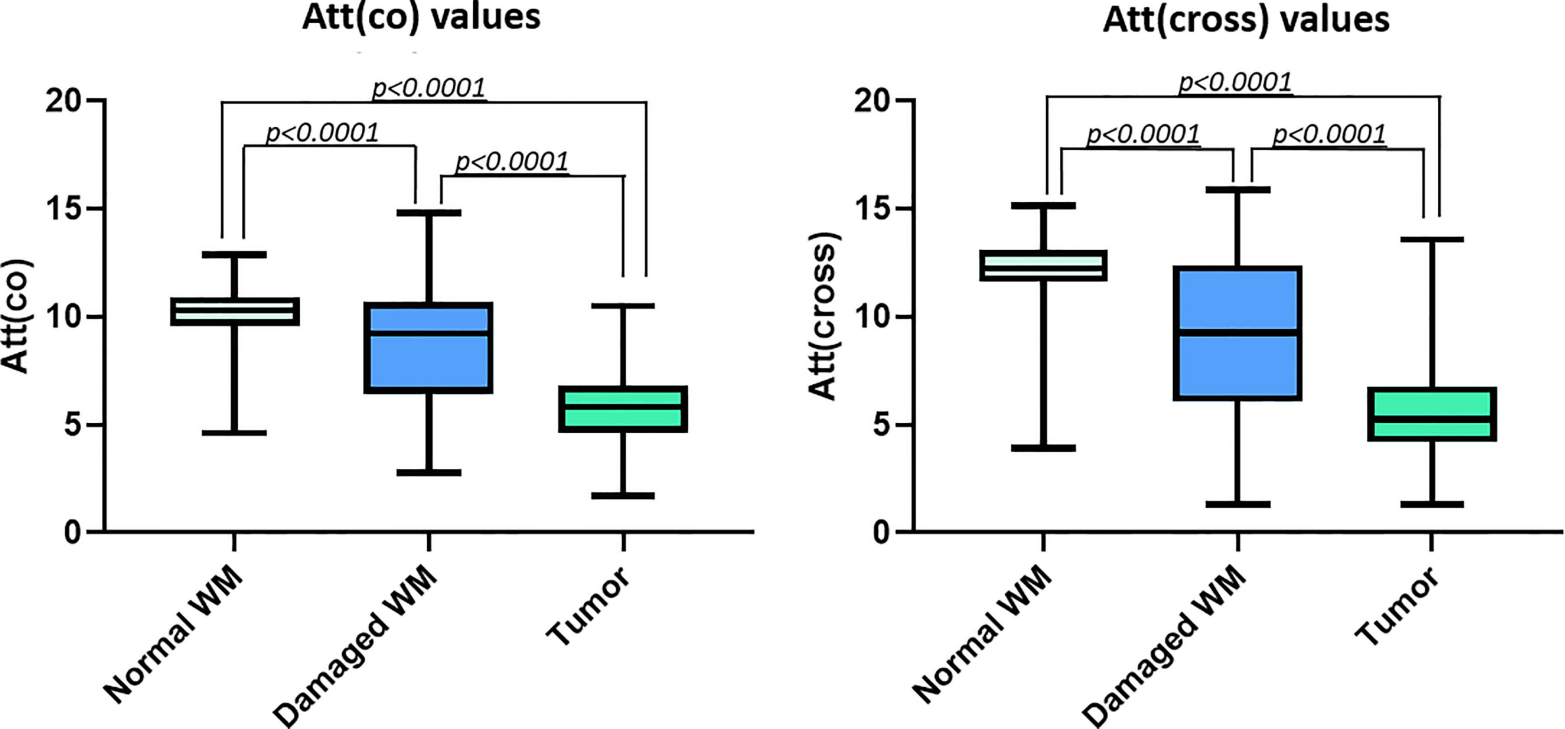
Distributions of Att(co) and Att(cross) values for the studied tissue types.

**Figure 4:**
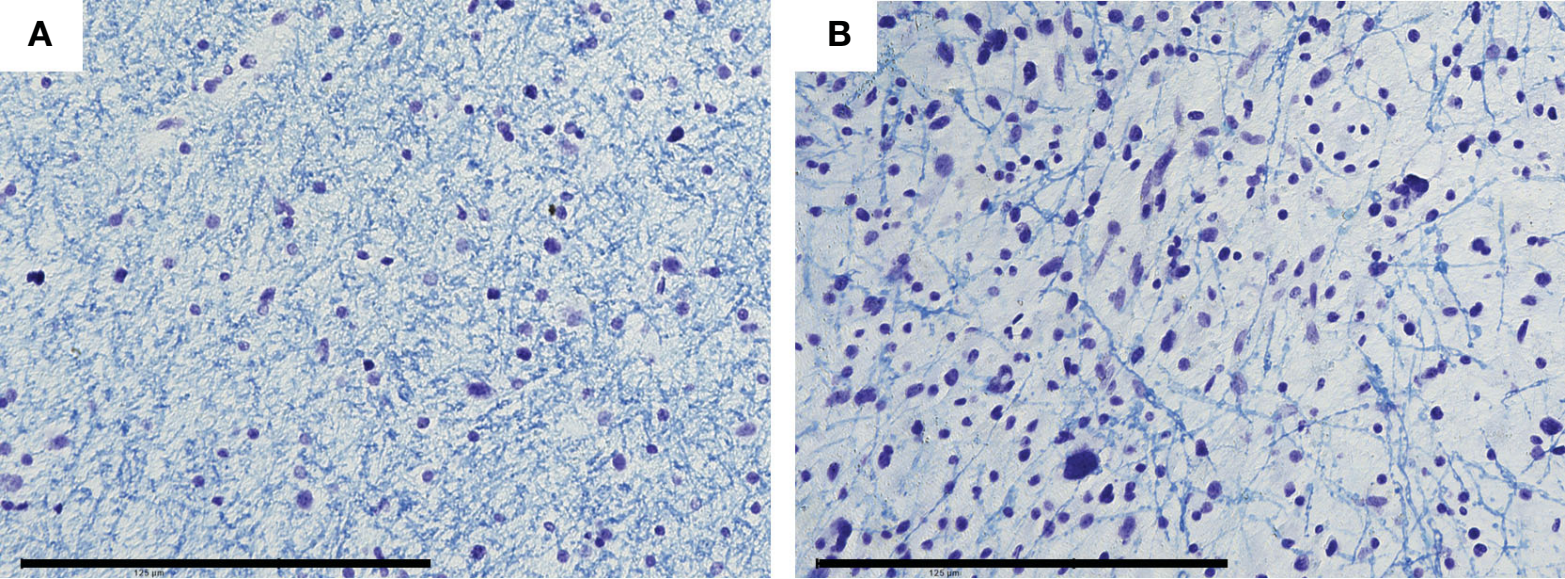
Morphological heterogeneity of damaged white matter is predominantly based on different amount of preserved myelinated fibers that varies from formed grid, which is sparser compared to areas of normal white matter **(A)** to areas with individual preserved fibers **(B)**. Scale bar = 200 µm.

Quantitative processing of the OCT signal using attenuation coefficients further confirms differences between all the studied types of tissues with high accuracy (**Tables 2**, **3**). Normal white matter is characterized by highest values of the coefficients in co- and cross-polarizations, while the scattering properties of the tumor tissue are significantly reduced. It was found that the destruction of myelinated fibers in the region of interest leads to deterioration in scattering properties, which is reflected in a decrease in the attenuation coefficients values in both polarizations compared to normal white matter. Thus, the white matter, characterized by the destruction of myelinated fibers in the study area, occupies an intermediate position between normal white matter and tumor tissue (**Figure 3**). At the same time, it is worth mentioning that, in contrast to normal white matter and a tumor, there is a greater variability of the values of both attenuation coefficients for damaged white matter, which is associated with morphological heterogeneity of samples, in particular, with a different amount of altered myelinated fibers in the studied area (**Figure 4**).

**Table 2:** Comparison of the studied tissue types using Att(co) coefficient.

	Normal white matter (n=169) 10.3 [9.6; 10.9]^a^	Damaged white matter (n=407) 9.2 [6.4; 10.7]^a^	Tumor (n=297) 5.8 [4.6; 6.8]^a^
**Normal white matter**	–	<0.0001	<0.0001
**Damaged white matter**	<0.0001	–	<0.0001
**Tumor**	<0.0001	<0.0001	–

p-values for the alternative hypothesis of the Mann-Whitney U-test about the presence of differences between the compared groups are indicated.

a Me [Q1;Q3] – where Me – median, Q1, Q3 – values of 25^th^ and 75^th^ percentiles, respectively.

**Table 3:** Comparison of the studied tissue types using Att(cross) coefficient.

	Normal white matter (n=169) 12.2 [11.6; 13.0]^a^	Damaged white matter (n=407) 9.2 [6.0; 12.3]^a^	Tumor (n=297) 5.3 [4.2; 6.8]^a^
**Normal white matter**	–	<0.0001	<0.0001
**Damaged white matter**	<0.0001	–	<0.0001
**Tumor**	<0.0001	<0.0001	–

p-values for the alternative hypothesis of the Mann-Whitney U-test about the presence of differences between the compared groups are indicated.

a Me [Q1;Q3] – where Me – median, Q1, Q3 – values of 25^th^ and 75^th^ percentiles, respectively.

Correlation analysis performed using Spearman’s rank correlation coefficient demonstrated statistically significant negative correlation between both attenuation coefficients and morphological types of brain tissue that were encoded as “1” – normal white matter, “2” – damaged white matter, “3” – tumor (**Table 4**). Thus, the lower values of attenuation coefficient correspond to more pathologically altered tissue state.

**Table 4:** The results of correlation analysis of optical coefficients and brain tissue types.

	Spearman’s rank correlation coefficient	p-value
**Att(co)**	-0,5909	<0,0001
**Att(cross)**	-0,6047	<0,0001

However, in view of the morphological heterogeneity of the studied samples, the usage of individual numerical value obtained from CP OCT image may be insufficient. Thereby, we decided to carry out visual analysis of color-coded maps, representing the optical coefficients’ values distribution throughout the OCT image.

### 3.3 Application of color-coded optical maps for brain tissue type differentiation

**Figure 5:**
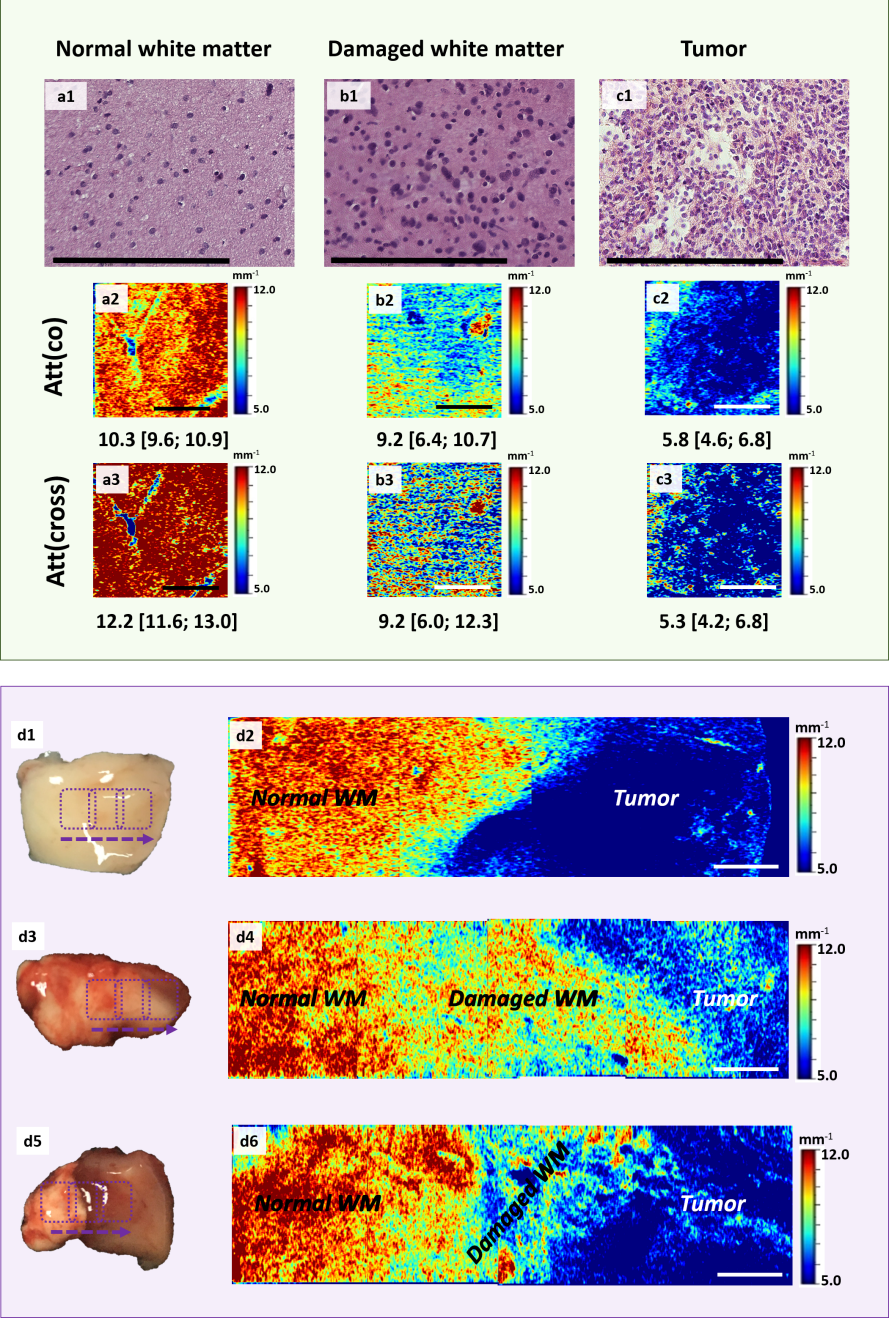
Application of color-coded maps for brain tissue visualization. Examples of en-face color-coded maps built based on the Att(co) **(a2, b2, c2)** and Att(cross) **(a3, b3, c3)** values distributions for all the studied tissue types with corresponding histology in H&E staining **(a1, b1, c1)**. **(d2, d4, d6)** – color-coded map of Att(co) representing different examples of tumor border where the areas of damaged and normal white matter can be visualized. The border between normal white matter and tumor may vary from a narrow strip of damaged white matter **(d2)** to a broad zone with smaller **(d4)** or bigger **(d6)** amount of altered myelinated fibers. The area of scanning is marked on the sample **(d1, d3, d5)** using purple rectangles. Scale bar = 200 µm on histological image and 1 mm on OCT image.

Third stage of the study included visual analysis of color-coded maps representative for three studied tissue types. Optical maps allow to present data in a customizable color palette. In previous studies, optical maps were used to distinguish white matter and tumor where areas with high attenuation coefficients (normal white matter) were presented dominantly by bright hues (orange to deep red), while low optical coefficients (tumor) were rendered in cyan and blue (13). In the present work, for the first time we developed optical maps also representing damaged white matter. As **Figures 5b2, b3** demonstrates, the destruction of myelinated fibers leads to the predominance of intermediate colors on optical maps, in particular, green and yellow. In addition, these maps are more heterogeneous, characterized by the presence of areas with high attenuation coefficients (areas with preserved myelin fibers) and zones with low values of these coefficients (total fiber destruction).

Thus, we distinguished the following characteristics of optical maps representing the distributions of co- and cross-polarization attenuation coefficients for three studied tissue types:

A) normal white matter (**Figures 5a1–a3**)

- Att(co): Total prevalence of dark red and orange. In rare cases: presence of yellow

- Att(cross): Total prevalence of dark red color

B) damaged white matter (**Figures 5b1–b3**)

- Att(co): The most heterogeneous group; prevalence of azure, green and yellow colors; possible presence of blue and red areas

- Att(cross): Multicolored maps; possible presence of all colors: red, yellow, green, azure, blue

C) tumor (**Figures 5c1–c3**)

- Att(co): Total prevalence of blue; in rare cases: presence of areas with higher values of Att(co): azure, green, yellow

- Att(cross): Total prevalence of blue color; in rare cases: presence of areas with higher values of Att(cross): azure, yellow, red

Importantly, in certain cases differentiation of areas of damaged white matter from both tumor and normal white matter is complicated due to overlap of attenuation coefficient values for differentiable tissue types. In particular, with a small amount of damaged myelin fibers in the study area, the values of the coefficients decrease moderately and are represented in yellow. At the same time, areas of normal white matter are also characterized by presence of yellow color in rare cases. Areas of total fiber destruction are characterized by the appearance of azure-blue hues, which can be confused with a tumor (**Figures 5d1–d6**).

### 3.4 The diagnostic ability of visual assessment of structural CP OCT images and color-coded maps to differentiate various tissue types in the peritumoral area

To assess the possibility of using CP OCT as a neuronavigation method during brain tumors resections we evaluated the level of diagnostic accuracy of visual analysis of structural CP OCT images and optical maps.

Our study demonstrates that both approaches, visual assessment of CP OCT images as well as use of color-coded maps, allow researchers to differentiate three types of studied brain tissue from each other. The assessed inter-rater agreement revealed a higher level of agreement between all the respondents in the case of evaluation of color-coded maps (k = 0.79 and k = 0.77 for Att(co) and Att(cross) maps, respectively) compared to the analysis of B-scans (k=0.64). We suggest it is caused by greater clarity of the data presented in optical maps. In this case, the respondents had to analyze only presence and predominance of different colors (blue, azure, green, yellow, orange and red) in contrast to structural CP OCT images, where three parameters were needed to be assessed.

**Figure 6:**
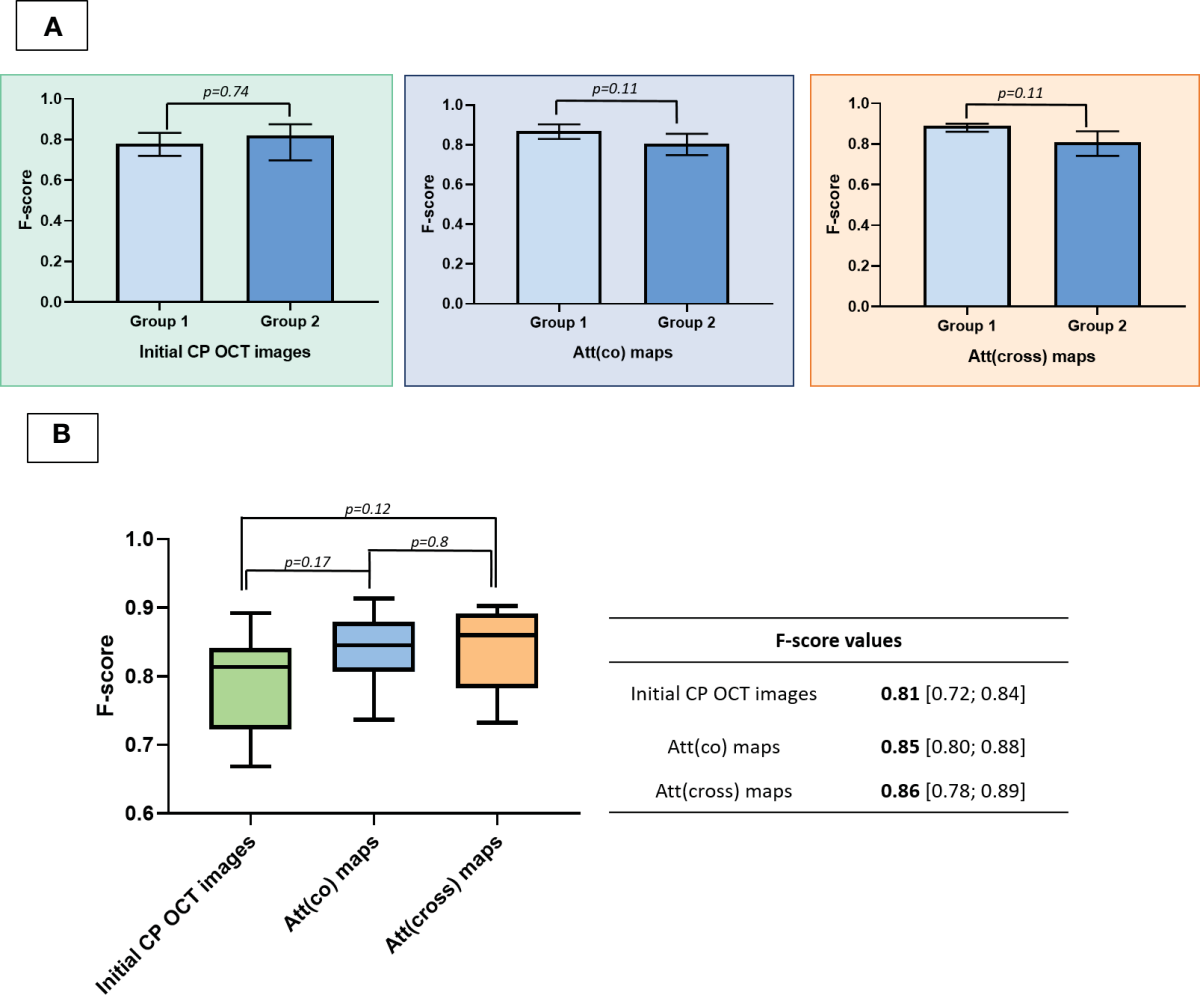
Diagnostic ability of different approaches to OCT data assessment for delineation of brain tissue types in the peritumoral area. **A–F**-score values of using different approaches to the evaluation of CP OCT images to differentiate normal white matter, damaged white matter and tumor. **B–F**-score values calculated in 2 subgroups of respondents where “Group 1” includes researchers experienced in working with OCT data and “Group 2” consists of researchers without previous experience in the analysis of OCT data. Data is presented as Me [Q1; Q3].

As mentioned above, the diagnostic accuracy of the image interpretation test was assessed using the F-score parameter due to three answer options being available in the test. Importantly, the F-score values obtained in the two groups of respondents did not differ significantly from each other (**Figure 6A**). This could be explained by high-quality training of the second group of respondents (biomedical researchers with no experience in working with OCT data) before passing the test. In this way, we suppose that with an adequate training visual analysis of structural CP OCT images and optical maps may be carried out by a researcher or a doctor without the additional help of an OCT specialist. In light of us not finding differences between the groups of respondents, further experiment in identifying differences in the diagnostic accuracy of the visual assessment of B-scans and optical maps was conducted on a combined group of respondents, which included all the specialist participants.

F-score values were initially calculated for each respondent separately, resulting in three values for each respondent, corresponding to the analysis of B-scans, Att(co) maps, and Att(cross) maps. Subsequently, all the values were presented in one table with the F-score values for each respondent in the rows and the type of images being evaluated in the corresponding columns. Afterwards, we analyzed the diagnostic accuracy level of used approaches to OCT data analysis. It has been demonstrated that the use of color-coded maps improves the diagnostic accuracy of the method (0.85-0.86 compared to 0.81) and provides objective information about the scattering properties of brain tissue (**Figure 6B**).

As it was mentioned above, we expected that the additional use usage of cross-polarization coefficient would provide us with more information about the morphological features of studied brain tissues. However, we have not discovered any additional advantages in using Att(cross) coefficient. Therefore, in future studies and clinical practice it could be sufficient to use Att(co) coefficient for visualization and differentiation brain tissue types in the peritumoral zone.

## 4 Discussion

In recent decades, studies on brain tissue imaging using OCT have mainly focused on the differentiation between normal and tumor tissues. There are several studies demonstrating the possibility of distinguishing normal white matter from tumor based on visual analysis of structural OCT images and quantitative signal processing using the attenuation coefficient and optical maps (14, 16, 17, 28). In addition, some studies go further to apply machine learning and artificial intelligence for the purpose of tissue differentiation (18, 29). However, none of the studies have paid close attention to the peritumoral area in general, and the issue of white matter morphological features in particular, which is important for improving the quality of tumor resections.

In the present work, for the first time we carried out a targeted assessment of the scattering properties of peritumoral white matter, characterized by damage to myelinated fibers using CP OCT. In addition, we evaluated the diagnostic ability of visual assessment of structural CP OCT images and color-coded maps of Att(co) and Att(cross) to differentiate normal white matter, damaged white matter and tumor.

We determined the visual features of structural CP OCT images, as well as color-coded optical maps, characteristic of each of the studied tissue types. Additionally, the median values of the attenuation coefficients in co- and cross-polarizations were calculated. Subsequently, two groups of respondents were offered to pass two classification tests (containing sets of B-scans and optical maps), the results of which determined the level of diagnostic accuracy of the method.

During the study, it was found that areas of damaged white matter are characterized by a decrease in scattering properties in both polarizations compared to the tissue of normal pathways. This phenomenon can be detected both by visual analysis of structural OCT images and by applying quantitative data processing followed by analysis of color-coded optical maps.

It should be noted that areas of damaged white matter are structurally heterogeneous because the tumor has a complex effect on the tissue of the pathways (4). On the one hand, there is a destruction of myelinated nerve fibers, the exact mechanism of which has not yet been established (30). In our previous work (31), we carried out a quantitative assessment of the relationship between the morphological and optical properties of normal white matter, and it was shown that myelinated fibers make the main contribution to its scattering properties. In the present work, we found that differences in the amount of damaged myelinated fibers in the studied samples result in a large variability of the attenuation coefficients values compared with areas of normal white matter. Color-coded optical maps of these samples are also heterogeneous due to the content of areas with a large number of preserved myelin fibers, as well as areas with their complete destruction. However, the destruction of myelinated fibers is not the only consequence of the influence of the tumor on the tissue of the white matter. At the same time, areas of damaged white matter are characterized by infiltration by tumor cells, as well as the occurrence of vasogenic edema (32). Thus, in each patient diagnosed with a brain neoplasm, complex changes in the structural characteristics of the white matter occur in comparison with the normal state. At the same time, in each specific case, a unique combination is observed, consisting in a different degree of edema and in a different amount of damaged myelinated fibers. In this regard, scattering properties only cannot precisely reflect the percentage of damaged myelin fibers in each particular case.

In addition, we analyzed the diagnostic ability of the method to differentiate three types of tissue in the peritumoral zone. It should be noted that the introduction of a third type of tissue, characterized by an intermediate position between normal white matter and a tumor, leads to a decrease in the diagnostic accuracy of the method, compared with the distinction between tumor and normal tissues only. For example, in the work of Yashin et al. (33), the diagnostic accuracy of visual analysis of structural OCT images for the differentiation of normal white matter and glial tumors was 87-88%. The need to detect areas with destroyed myelinated fibers complicates the study, which is reported to be due to the greater heterogeneity of OCT images obtained from this type of tissue. The use of quantitative processing of OCT data with threshold values of optical coefficients makes it possible to objectify the data and increase the diagnostic accuracy of the method, which was demonstrated by several groups (13, 14). However, during surgery, the assessment of a single numerical value obtained from an OCT image may not be sufficient, in particular, if several types of tissue are included in the field of view. In this regard, the use of color-coded optical maps looks more promising for distinguishing between normal and pathological brain tissues. This approach combines both the clarity of the visual assessment of structural OCT images and the objectivity of quantitative data processing. We demonstrate that the analysis of optical maps allows a slight increase in the diagnostic accuracy of the method, compared with the evaluation of structural OCT images (F-score = 0.85-0.86 and 0.81 for the assessment of optical maps and structural OCT images, respectively). In addition, it has been shown that optical maps allow presenting the data in a more accessible form for respondents in comparison with B-scans, which is reflected in the level of inter-rater agreement. Moreover, it is interesting that we did not find a significant advantage in respondents who work daily with OCT images and, accordingly, have significant experience in “reading” them. This fact demonstrates the prospects for the use of visual analysis of CP OCT data intraoperatively by the neurosurgeon without additional specialists.

The use of numerical values of the attenuation coefficients also makes it possible to distinguish three types of tissue from each other with high accuracy (p<0.0001). In this regard, the additional use of median values of the attenuation coefficients in the OCT image can be useful in cases where predominated colors on the optical maps, reflect the cross values of the coefficients between adjacent tissue types (normal white matter/damaged white matter or damaged white matter/tumor). Thus, for intraoperative determination of tissue type, it looks promising to build optical maps with simultaneous calculation of the median value of the optical coefficient (clinical example demonstrated in **Supplementary Figure 4**).

In view of applying our results in clinical practice, it is worth noting that this study was carried out on ex vivo samples of brain tissue. We assume that transportation of samples in closed Petri dishes on ice preserves the structural characteristics and, consequently, the optical properties of the object and the results of the experiment demonstrated (34). However, to confirm the obtained results, it is necessary to carry out *in vivo* studies during surgical intervention.

Moreover, study limitations connected with several aspects are needed to be marked. On the one hand, we need to mention the limitations of OCT method, in particular, low penetration depth of the probing light. Therefore, it is possible to obtain the information about the tissue structure only of the depth up to 1.5 mm. This aspect also includes the small size of the OCT image and, accordingly, the small volume of tissue scanning. On the other hand, during the evaluation of myelinated fibers preservation in the study area we cannot exclude the influence of edema on the features of the obtained OCT signal. It is known, that brain tissue edema causes differences in its scattering properties (35, 36). Consequently, the severe edema may significantly decrease the scattering properties of white matter, which may be confusing in the case of low amount of destructed myelinated fibers. In addition, the areas of coagulation, hemorrhages and necrosis may also lead to changes in the nature of the received OCT signal, which is important especially if we are speaking about in vivo studied during surgery.

To summarize, OCT is a promising tool for neuronavigation during resection of malignant neoplasms of the brain or stereotaxic biopsies. Currently, various options for intraoperative OCT systems are known (integration into an operating microscope or the use of optical probes) (20, 37), which indicates the possibility of intraoperative application of this method to obtain precise information about the brain tissue type in a specific region of interest.

## 5 Conclusions

We discovered that alteration of myelinated fibers causes changes in the scattering properties of the white matter and OCT is a promising tool for studying the state of the white matter for the subsequent differentiation of tissue types in the perifocal zone of the tumor. To accomplish this task, it is possible to use both visual analysis of structural OCT images and the use of optical maps. The construction of color-coded maps makes it possible to objectify the information, maintaining the visibility of the visual assessment, while there is an increase in diagnostic accuracy (F-score = 0.85-0.86 and 0.81 for the assessment of optical maps and structural OCT images, respectively). At the same time, the presence of prior experience with OCT images does not provide an advantage in the image classification process. Thus, data analysis will not require the participation of a specially trained person and can be carried out directly by a neurosurgeon after the adequate training. In addition, we have not discovered any advantages of additional usage of cross-polarization, which demonstrates the ability to determine type of tissue in the peritumoral area using non-polarization OCT devices.

## Data availability statement

The raw data supporting the conclusions of this article will be made available by the authors, without undue reservation.

## Ethics statement

The studies involving human participants were reviewed and approved by Institutional Review Board of Privolzhsky Research Medical University. The patients/participants provided their written informed consent to participate in this study. Written informed consent was obtained from the individual(s) for the publication of any potentially identifiable images or data included in this article.

## Author contributions

KA: study concept and design, data acquisition and quality control of data, data analysis and interpretation, manuscript preparation. KY, EK, EB, ML, IM: data acquisition and quality control of data. AM: data analysis, manuscript preparation. EK, GG, EZ and NG: manuscript review. All authors contributed to the article and approved the submitted version.

## References

[1] Sung H Ferlay J Siegel RL Laversanne M Soerjomataram I Jemal A. Global cancer statistics 2020: GLOBOCAN estimates of incidence and mortality worldwide for 36 cancers in 185 countries. CA Cancer J Clin (2021) 71:209–49. doi: 10.3322/caac.21660 33538338

[2] Leece R Xu J Ostrom QT Chen Y Kruchko C Barnholtz-Sloan JS. Global incidence of malignant brain and other central nervous system tumors by histology, 2003-2007. Neuro Oncol (2017) 19(11):1553–64. doi: 10.1093/neuonc/nox091 PMC5737839 28482030

[3] Witwer BP Moftakhar R Hasan KM Deshmukh P Haughton V Field A. Diffusion-tensor imaging of white matter tracts in patients with cerebral neoplasm. J Neurosurg (2002) 97(3):568–75. doi: 10.3171/jns.2002.97.3.0568 12296640

[4] Yen PS Teo BT Chiu CH Chen SC Chiu TL Su CF. White matter tract involvement in brain tumors: a diffusion tensor imaging analysis. Surg Neurol (2009) 72(5):464–9. doi: 10.1016/j.surneu.2009.05.008 19608227

[5] Jackson C Westphal M Quiñones-Hinojosa A. Complications of glioma surgery. Handb Clin Neurol (2016) 134:201–18. doi: 10.1016/B978-0-12-802997-8.00012-8 26948356

[6] Romano A Fasoli F Ferrante M Ferrante L Fantozzi LM Bozzao A. Fiber density index, fractional anisotropy, adc and clinical motor findings in the white matter of patients with glioblastoma. Eur Radiol (2008) 18(2):331–6. doi: 10.1007/s00330-007-0740-9 17899109

[7] Szmuda T Kierońska S Ali S Słoniewski P Pacholski M Dzierżanowski J. Tractography-guided surgery of brain tumours: what is the best method to outline the corticospinal tract? . Folia Morphol (Warsz) (2021) 80(1):40–6. doi: 10.5603/FM.a2020.0016 32073136

[8] Henderson F Abdullah KG Verma R Brem S. Tractography and the connectome in neurosurgical treatment of gliomas: the premise, the progress, and the potential. Neurosurg Focus (2020) 48(2):E6. doi: 10.3171/2019.11.FOCUS19785 PMC7831974 32006950

[9] Gerard IJ Kersten-Oertel M Hall JA Sirhan D Collins DL. Brain shift in neuronavigation of brain tumors: An updated review of intra-operative ultrasound applications. Front Oncol (2021) 10:618837. doi: 10.3389/fonc.2020.618837 33628733 PMC7897668

[10] Roberts DW Valdes PA Harris BT Fontaine KM Hartov A Fan X. Coregistered fluorescence-enhanced tumor resection of malignant glioma: Relationships between delta-aminolevulinic acid-induced protoporphyrin IX fluorescence, magnetic resonance imaging enhancement, and neuropathological parameters. J Neurosurg (2011) 114:595–603. doi: 10.3171/2010.2.JNS091322 20380535 PMC2921008

[11] Sastry R Bi WL Pieper S Frisken S Kapur T Wells W. Applications of ultrasound in the resection of brain tumors. Neuroimaging (2017) 27(1):5–15. doi: 10.1111/jon.12382 PMC5226862 27541694

[12] Leitgeb R Placzek F Rank E Krainz L Haindl R Li Q. Enhanced medical diagnosis for dOCTors: a perspective of optical coherence tomography. J BioMed Opt (2021) 26(10):100601. doi: 10.1117/1.JBO.26.10.100601 34672145 PMC8528212

[13] Yashin KS Kiseleva EB Moiseev AA Kuznetsov SS Timofeeva LB Pavlova NP. Quantitative nontumorous and tumorous human brain tissue assessment using microstructural co- and cross-polarized optical coherence tomography. Sci Rep (2019) 9:2024. doi: 10.1038/s41598-019-38493-y 30765763 PMC6375924

[14] Kut C Chaichana KL Xi J Raza SM Ye X McVeigh ER. Detection of human brain cancer infiltration ex vivo and in vivo using quantitative optical coherence tomography. Sci Transl Med (2015) 7:292ra100. doi: 10.1126/scitranslmed.3010611 PMC4482228 26084803

[15] Wang H Akkin T Magnain C Wang R Dubb J Kostis WJ. Polarization sensitive optical coherence microscopy for brain imaging. Opt Lett (2016) 41(10):2213–6. doi: 10.1364/OL.41.002213 PMC5357322 27176965

[16] Yuan W Kut C Liang W Li. X. Robust and fast characterization of OCT-based optical attenuation using a novel frequency-domain algorithm for brain cancer detection. Sci Rep (2017) 7:44909. doi: 10.1038/srep44909 28327613 PMC5361149

[17] Bizheva K Unterhuber A Hermann B Povazay B Sattmann H Fercher AF. Imaging ex vivo healthy and pathological human brain tissue with ultra-high-resolution optical coherence tomography. J BioMed Opt (2005) 10:11006. doi: 10.1117/1.1851513 15847572

[18] Juarez-Chambi RM Kut C Rico-Jimenez JJ Chaichana KL Xi J Campos-Delgado DU. AI-Assisted *In situ* detection of human glioma infiltration using a novel computational method for optical coherence tomography. Clin Cancer Res (2019) 25:6329–38. doi: 10.1158/1078-0432.CCR-19-0854 PMC6825537 31315883

[19] Yashin K Bonsanto MM Achkasova K Zolotova A Wael AM Kiseleva E. OCT-guided surgery for gliomas: Current concept and future perspectives. Diagnostics (2022) 12:335. doi: 10.3390/diagnostics12020335 35204427 PMC8871129

[20] Böhringer HJ Lankenau E Stellmacher F Reusche E Hüttmann G Giese A. Imaging of human brain tumor tissue by near-infrared laser coherence tomography. Acta Neurochir (2009) 151(5):507–17. doi: 10.1007/s00701-009-0248-y PMC3085760 19343270

[21] Węglarczyk S. Kernel density estimation and its application. ITM Web Conferences (2018) 23:37. doi: 10.1051/itmconf/20182300037

[22] Gelikonov VM Romashov VN Shabanov DV Ksenofontov S Terpelov DA Shilyagin PA. Cross-polarization optical coherence tomography with active maintenance of the circular polarization of a sounding wave in a common path system. Radiophys Quant El (2018) 60:897–911. doi: 10.1007/s11141-018-9856-9

[23] Kiseleva EB Moiseev AA Kuyarov AS Molvi MA Gelikonov GV Maslennikova AV. *In vivo* assessment of structural changes of the urethra in lower urinary tract disease using cross-polarization optical coherence tomography. J Innov Opt Health Sci (2020) 13:2050024. doi: 10.1142/S1793545820500248

[24] Vermeer KA Mo J Weda JJ Lemij HG de Boer JF. Depth-resolved model-based reconstruction of attenuation coefficients in optical coherence tomography. BioMed Opt Express (2013) 5(1):322–37. doi: 10.1364/BOE.5.000322 PMC3891343 24466497

[25] Gubarkova EV Moiseev AA Kiseleva EB Vorontsov DA Kuznetsov SS Vorontsov AY. Tissue optical properties estimation from cross-polarization OCT data for breast cancer margin assessment. Laser Phys Lett (2020) 17(7):075602. doi: 10.1088/1612-202X/ab9091

[26] van der Velden E. CMasher: Scientific colormaps for making accessible, informative and'cmashing'plots. arXiv preprint arXiv (2020). 2003.01069. doi: 10.21105/joss.02004

[27] Liu Y Heer J. Somewhere over the rainbow: An empirical assessment of quantitative colormaps. Conf Hum Factors Comput Syst - Proc (2018), 1–12. doi: 10.1145/3173574.3174172

[28] Almasian M Wilk LS Bloemen PR van Leeuwen TG Ter Laan M Aalders MCG. Pilot feasibility study of *in vivo* intraoperative quantitative optical coherence tomography of human brain tissue during glioma resection. J Biophotonics (2019) 12(10):e201900037. doi: 10.1002/jbio.201900037 31245913 PMC7065626

[29] Möller J Bartsch A Lenz M Tischoff I Krug R Welp H. Applying machine learning to optical coherence tomography images for automated tissue classification in brain metastases. Int J Comput Assist Radiol Surg (2021) 16(9):1517–26. doi: 10.1007/s11548-021-02412-2 PMC8354973 34053010

[30] Brooks LJ Clements MP Burden JJ Kocher D Richards L Devesa SC. The white matter is a pro-differentiative niche for glioblastoma. Nat Commun (2021) 12:2184. doi: 10.1038/s41467-021-22225-w 33846316 PMC8042097

[31] Moiseev AA Achkasova KA Kiseleva EB Yashin KS Potapov AL Bederina EL. Brain white matter morphological structure correlation with its optical properties estimated from optical coherence tomography (OCT) data. BioMed Opt Express (2022) 13(4):2393–413. doi: 10.1364/BOE.457467 PMC9045907 35519266

[32] Cuddapah VA Robel S Watkins S Sontheimer H. A neurocentric perspective on glioma invasion. Nat Rev Neurosci (2014) 15(7):455–65. doi: 10.1038/nrn3765 PMC5304245 24946761

[33] Yashin KS Kiseleva EB Gubarkova EV Moiseev AA Kuznetsov SS Shilyagin PA. Cross-polarization optical coherence tomography for brain tumor imaging. Front Oncol (2019) 9:201. doi: 10.3389/fonc.2019.00201 31001471 PMC6455095

[34] Kiseleva EB Yashin KS Moiseev AA Sirotkina MA Timofeeva LB Fedoseeva VV. Cross-polarization optical coherence tomography in comparative *in vivo* and ex vivo studies of the optical properties of normal and tumorous brain tissues. Sovrem Tehnol v Med (2017) 9(4):177. doi: 10.17691/stm2017.9.4.22

[35] Liu J Li Y Yu Y Yuan X Lv H Zhao Y. Cerebral edema detection *in vivo* after middle cerebral artery occlusion using swept-source optical coherence tomography. Neurophotonics (2019) 6(4):45007. doi: 10.1117/1.NPh.6.4.045007 PMC6835117 31720312

[36] Rodriguez CL Szu JI Eberle MM Wang Y Hsu MS Binder DK. Decreased light attenuation in cerebral cortex during cerebral edema detected using optical coherence tomography. Neurophotonics (2014) 1(2):25004. doi: 10.1117/1.NPh.1.2.025004 PMC4321699 25674578

[37] Lankenau E Klinger D Winter C Malik A Müller HH Oelckers S. Combining optical coherence tomography (OCT) with an operating microscope. In: Buzug TM Holz D Bongartz J Kohl-Bareis M Hartmann U Weber S, editors. Advances in medical engineering. Springer, Berlin: Springer Proceedings in Physics (2007).

