# Supplementary material for: Nondestructive label-free detection of peritumoral white matter damage using cross-polarization optical coherence tomography
Source: Front Oncol. 2023 Mar 2;13:1133074. doi: 10.3389/fonc.2023.1133074 (PMC10017731; doi:10.3389/fonc.2023.1133074)
Supplement: Supplementary file 1 [file Presentation_1.pdf]

Supplementary Material

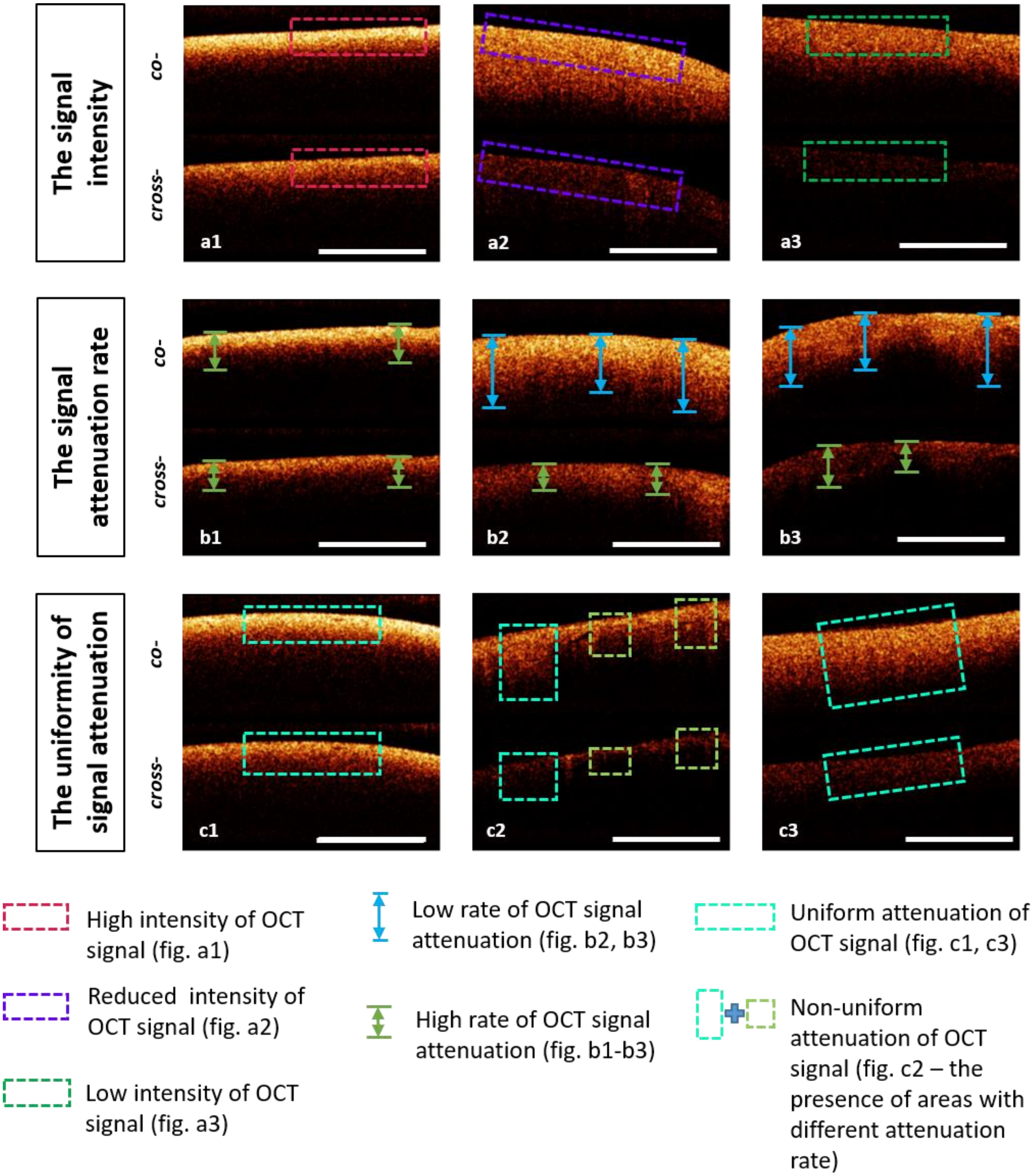

Supplementary Figure 1. Illustrations of CP OCT images with estimated parameters for visual assessment used in the training set. Scale bar – 1 mm.

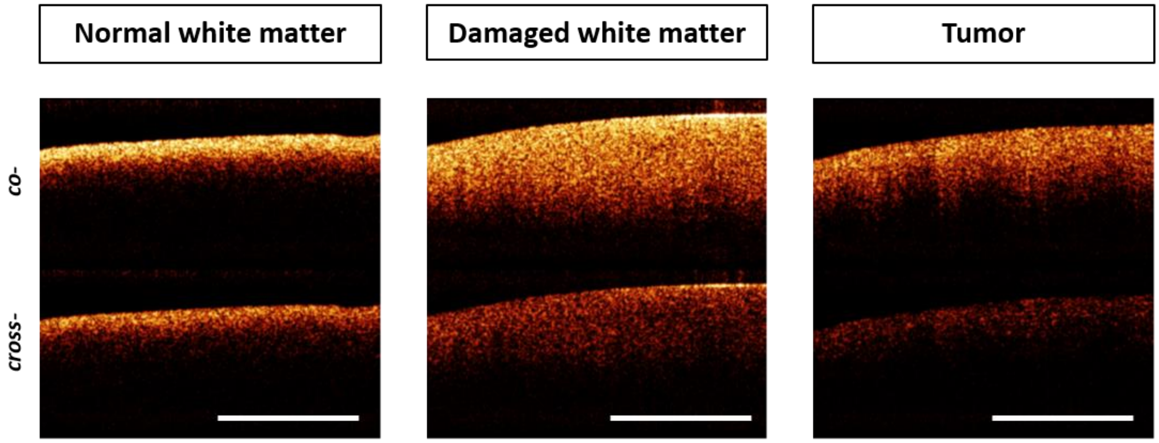

**The signal intensity** (the assessment is performed in both polarizations simultaneously)	High in co- and cross-polarizations	Reduced in co- and cross-polarizations (in cross-polarization decreases more pronouncedly) in comparison with normal white matter	Low in co- and cross-polarizations
**The signal attenuation rate** (the assessment is performed only in co-polarization)	High in co-polarization	Low in co-polarization	Low in co-polarization
**The uniformity of signal attenuation** (the assessment is performed in both polarizations simultaneously)	Uniform in co- and cross-polarizations	Uniform in co- and cross-polarizations	Non-uniform in co- and cross-polarizations

Supplementary Figure 2. Examples of images from the training set with classification criteria established for each studied tissue type.

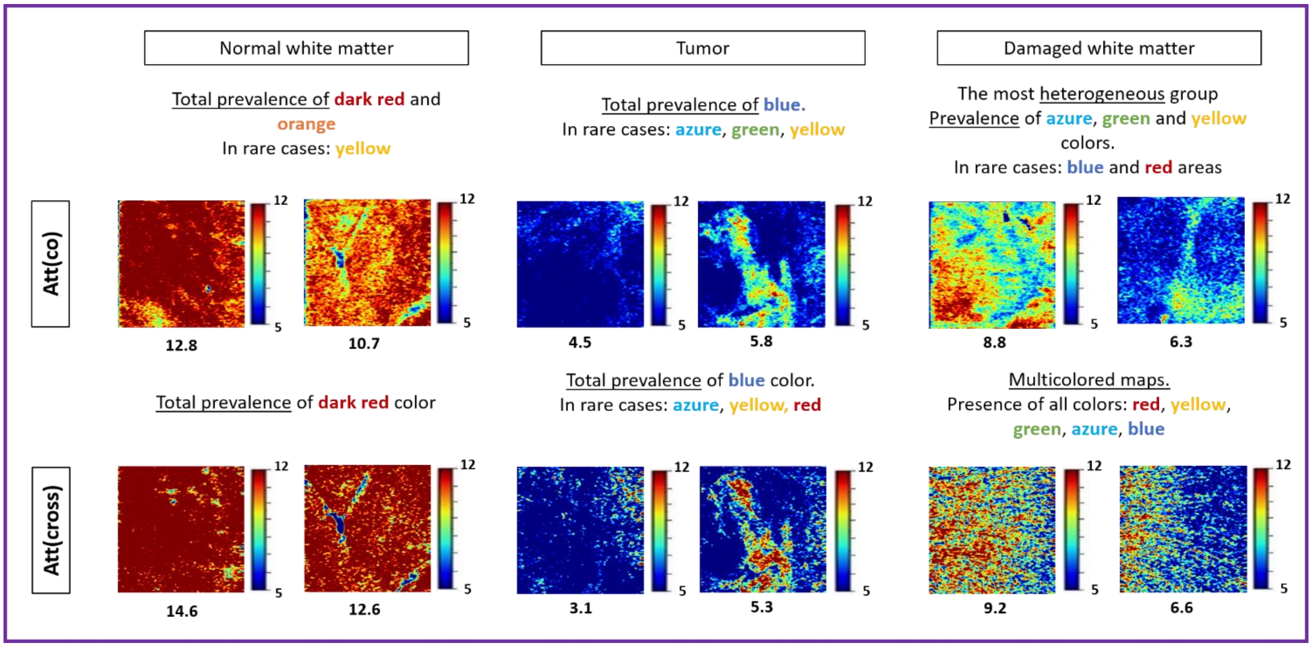

Supplementary Figure 3. The training set with classification criteria of optical maps established for each studied tissue type.

Clinical example of using CP OCT during tumor resection

Patient K., 31 years old. Case history includes recurrent episodes of headache, as well as visual hallucinations. MRI revealed an anaplastic astrocytoma (Grade III, IDH-mutant) in the left temporal lobe (Figure 4, a1, a2, a3), located near Wernicke's area. Surgical intervention with intraoperative awakening was performed to determine speech zones, and then the temporal lobe was isolated within anatomical landmarks. When the access to the tumor was carried out, the tissue sample was taken in the immediate vicinity of the tumor focus for the subsequent OCT scanning. Obtained images revealed features that are typical for damaged white matter (Figure 4, b3), namely: a homogeneous high-intensity signal with a low attenuation rate in both polarizations on structural OCT images; color-coded maps show intermediate values for both attenuation coefficients, being lower than those from normal white matter but higher than those from the tumor. After removal of the tumor lesion in the region of the posterior resection margin (Figure 4, b2, green dotted line), a sample of brain tissue was taken, which optical properties were distinctive for normal white matter (Figure 4, b4). According to postoperative MRI (Figure 4, c1,c2), total removal of the tumor was achieved.

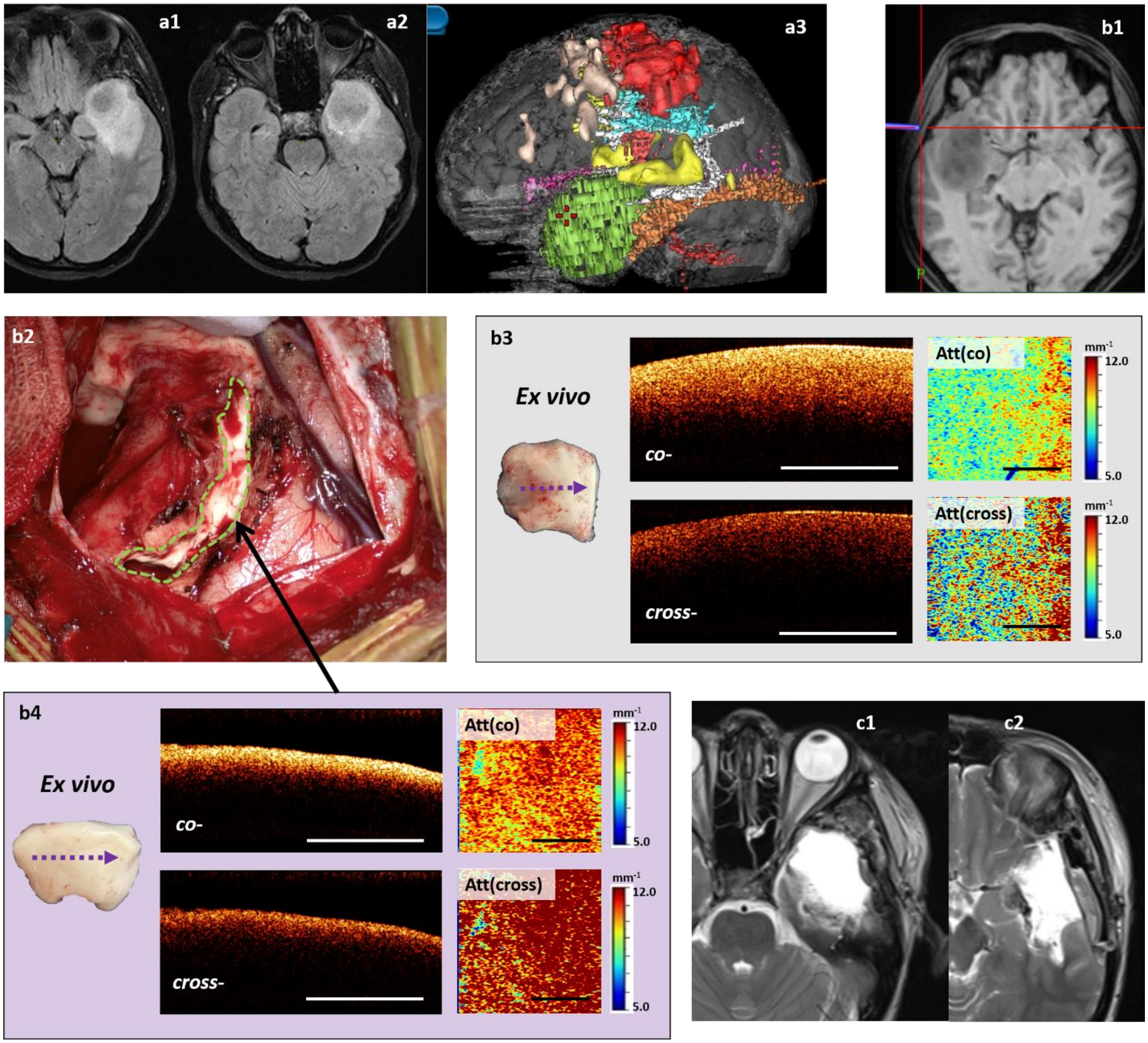

Supplementary Figure 4. The example of using OCT imaging of brain tissue samples obtained from patient during resection of anaplastic astrocytoma (Grade III, IDH-mutant) in the left temporal lobe. a1, a2, a3 – preoperative MRI images. During surgery using intraoperative neuronavigation (b1, b2), tissue sample was taken in the immediate vicinity of the tumor focus. Obtained OCT images revealed features that are typical for damaged white matter (b3). Imaging of the tissue sample obtained from the posterior resection margin demonstrated the signal characteristic of normal white matter (b4). The morphological characteristics of the collected samples were confirmed by histological examination. c1, c2 - postoperative MRI images, according to which total removal of the tumor was achieved.
